# Supplementary material for: Evaluation of a Serum-Free Medium for Human Epithelial and Stromal Cell Culture
Source: Int J Mol Sci. 2022 Sep 2;23(17):10035. doi: 10.3390/ijms231710035 (PMC9455993; doi:10.3390/ijms231710035)

Figure S1A: Morphology of KC at passages 1, 2 and 3.

Morphology of keratinocytes after four days (P1 d4: passage one, day 4), 11 days (P2 d4: passage two, day 4) and 18 days (P1 d4: passage three, day 4) of culture in serum-free medium or fetal bovine serum-containing medium. Experiments were performed in triplicate, and a representative image is shown. The images were captured at 10X magnification with a scale bare of 100µm.

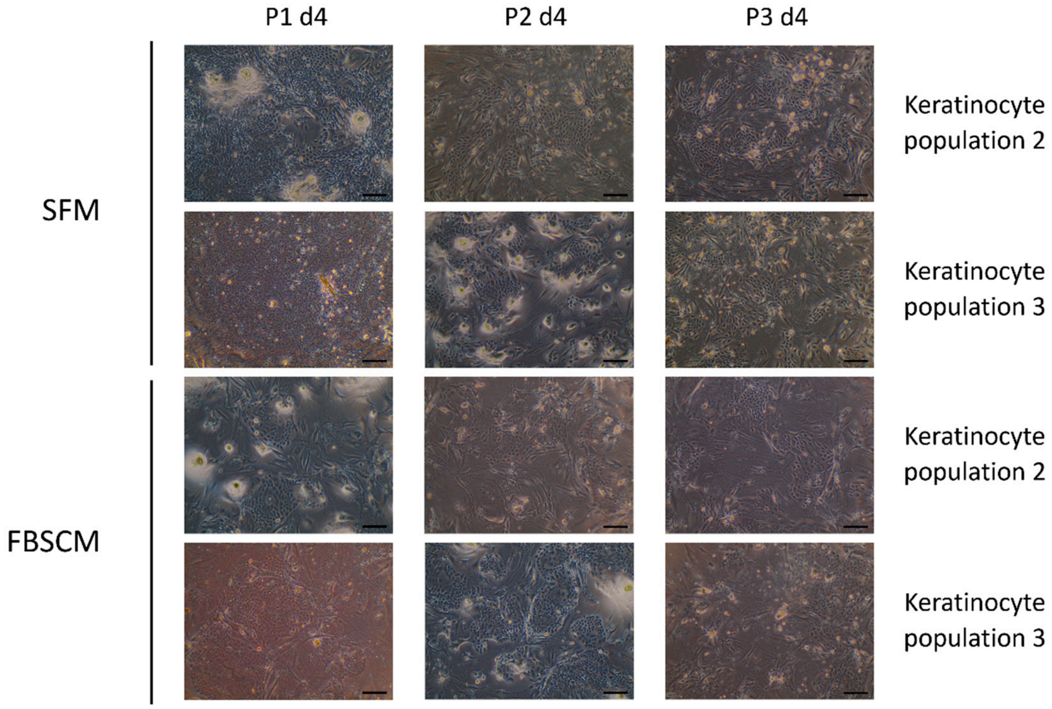

Figure S1B: Morphology of UC at passages 1, 2 and 3.

Morphology of urothelial cells after four days (P1 d4: passage one, day 4), 11 days (P2 d4: passage two, day 4) and 18 days (P1 d4: passage three, day 4) of culture in serum-free medium or fetal bovine serum-containing medium. Experiments were performed in triplicate, and a representative image is shown. The images were captured at 10X magnification with a scale bare of 100µm.

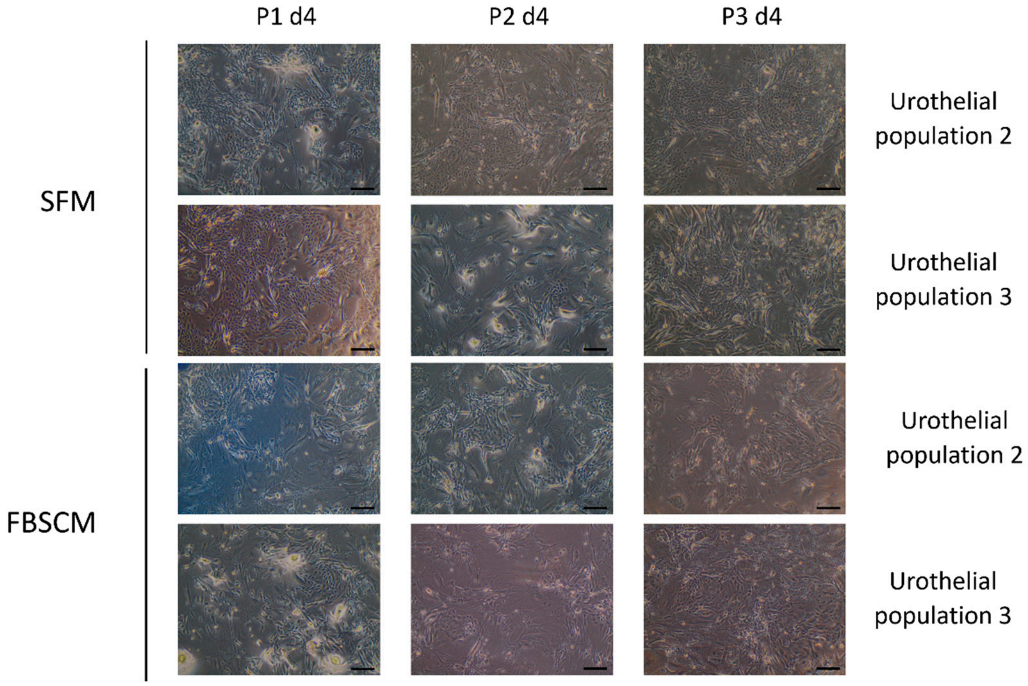

Figure S1C: Morphology of DF at passages 1, 2 and 3.

Morphology of dermal fibroblasts after four days (P1 d4: passage one, day 4), 11 days (P2 d4: passage two, day 4) and 18 days (P1 d4: passage three, day 4) of culture in serum-free medium or fetal bovine serum-containing medium. Experiments were performed in triplicate, and a representative image is shown. The images were captured at 10X magnification with a scale bare of 100µm.

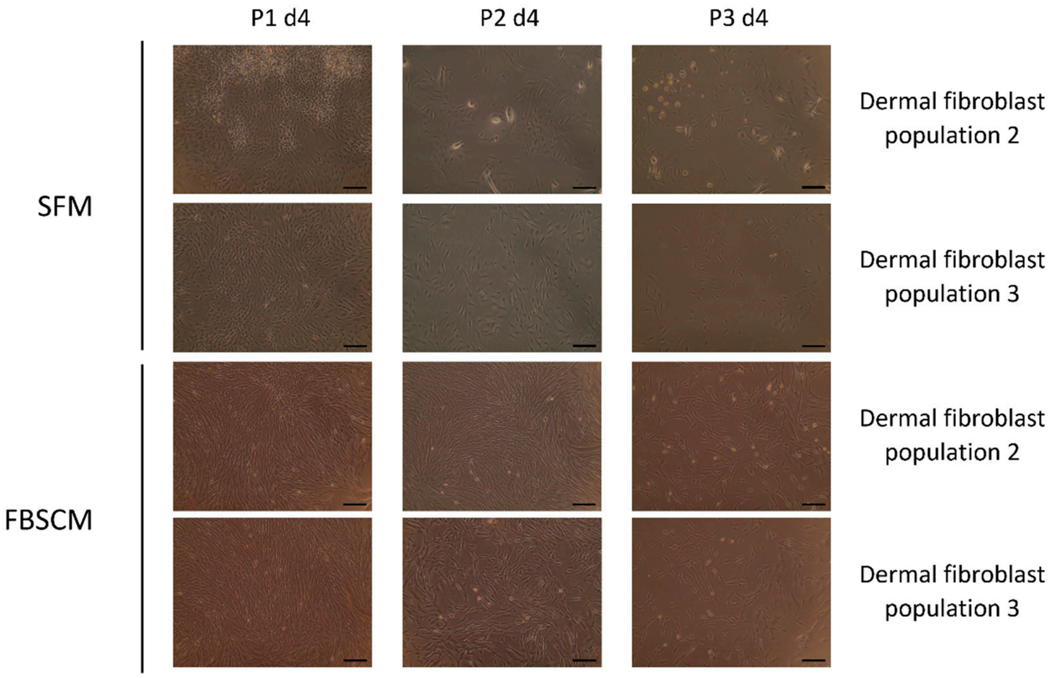

Figure S1D: Morphology of DF at passages 1, 2 and 3.

Morphology of vesical fibroblasts after four days (P1 d4: passage one, day 4), 11 days (P2 d4: passage two, day 4) and 18 days (P1 d4: passage three, day 4) of culture in serum-free medium or fetal bovine serum-containing medium. Experiments were performed in triplicate, and a representative image is shown. The images were captured at 10X magnification with a scale bare of 100µm.

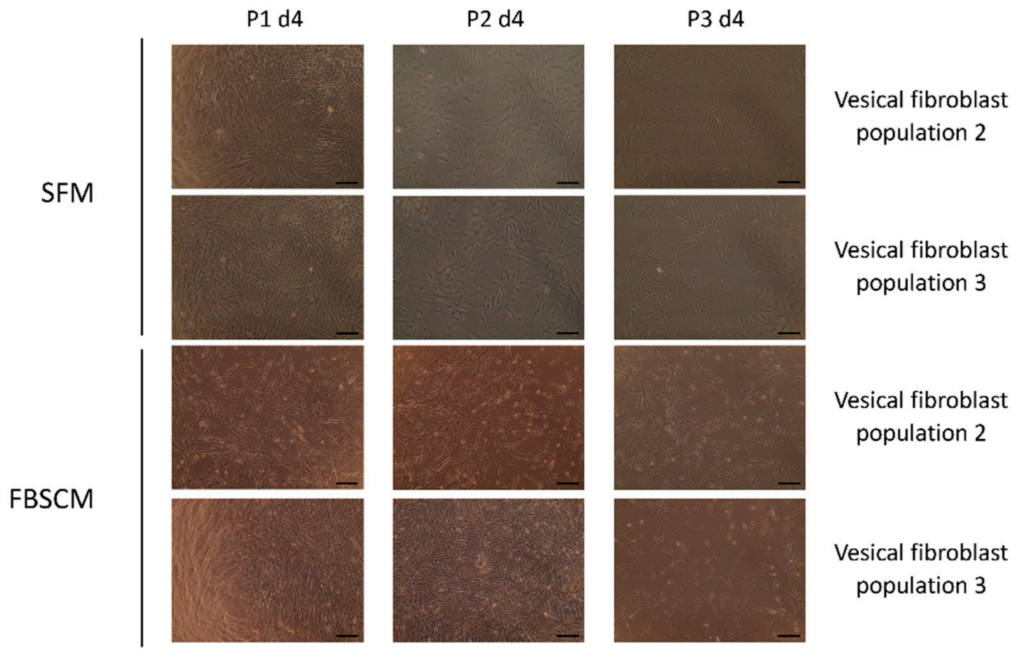

Figure S2A: Cell size evaluation of epithelial cells.

Cell size evaluation of keratinocytes (KC) and urothelial cells (UC) each day for three passages. Each dot represents the mean cell size of a cellular population realized in triplicate (N=3, n=3).

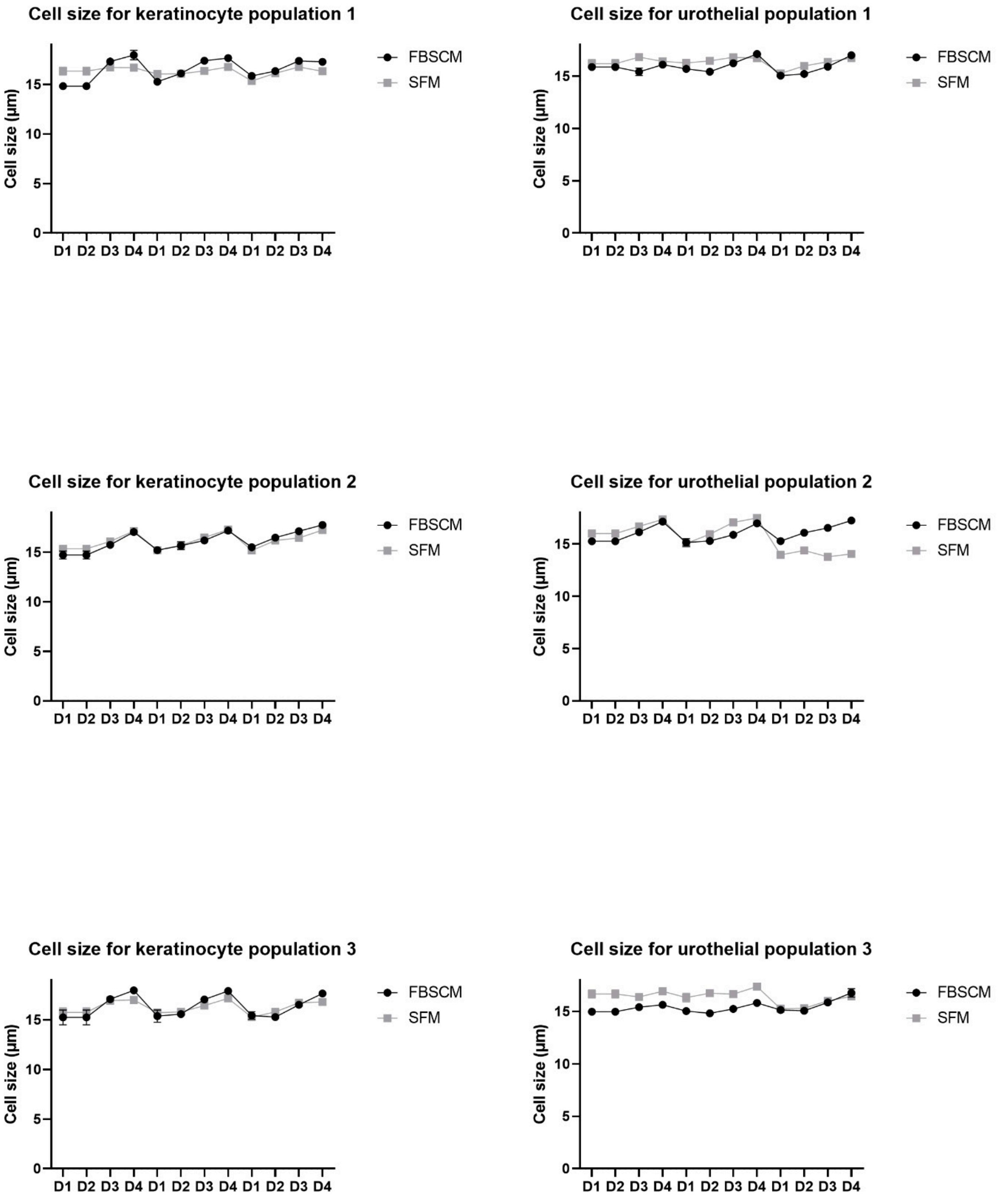

Figure S2B: Cell size evaluation of fibroblast cells.

For three passages, the cell size evaluation of dermal fibroblasts (DF) and vesical fibroblasts (VF) each day. Each dot represents the mean cell size of a cellular population realized in triplicate (N=3, n=3).

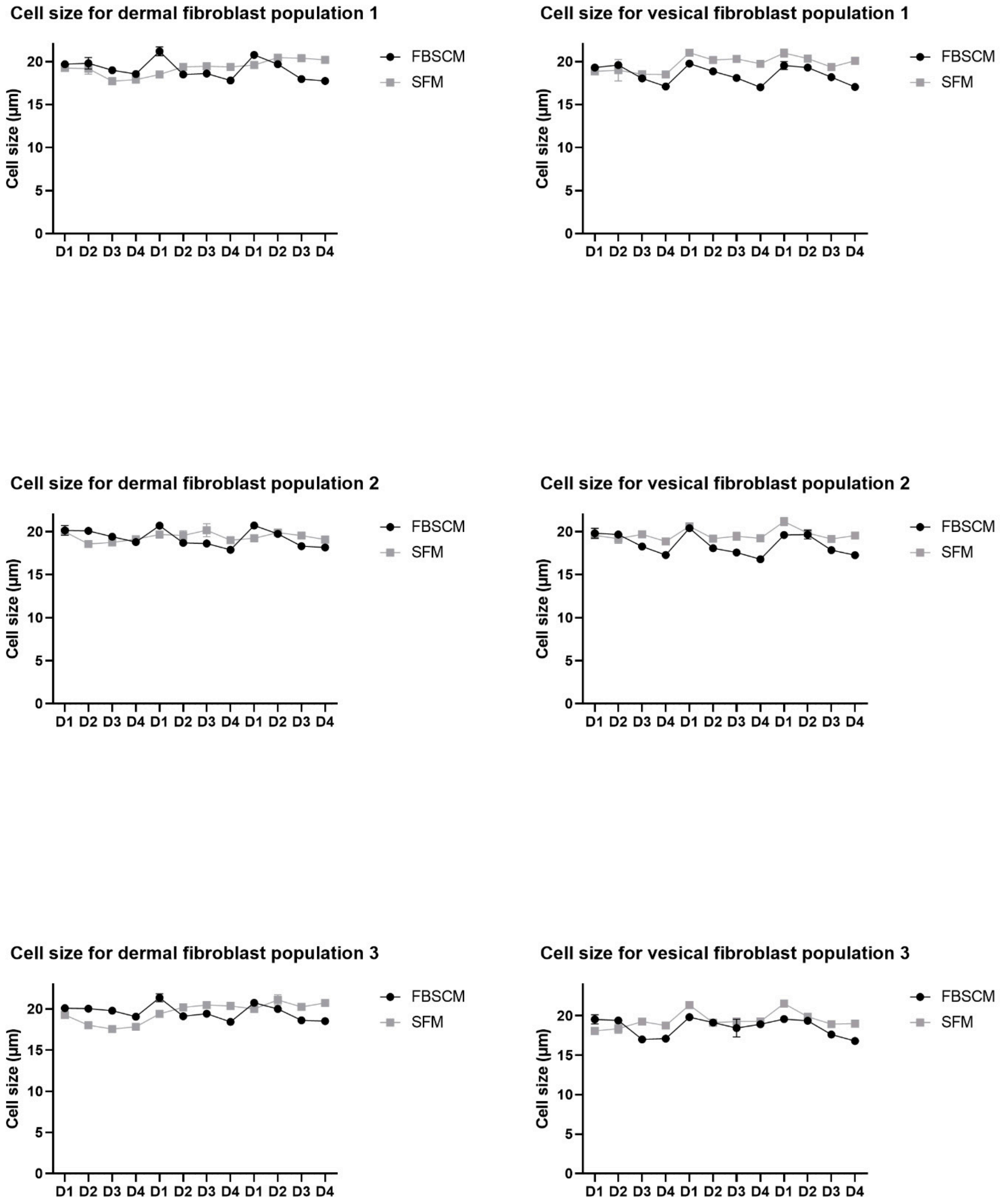

Figure S3A: Doubling time of epithelial cells at passages 1, 2 and 3.

Doubling time of epithelial cells over three passages. Ordinary two-way ANOVA has been used to interpret the data. Asterisks indicate significant differences: (*) for p-value<0.05, (**) for p-value<0.01. Each dot represents the mean of a cellular population realized in triplicate (n=3). Three points are presented as the experiment was done with three populations (N=3).

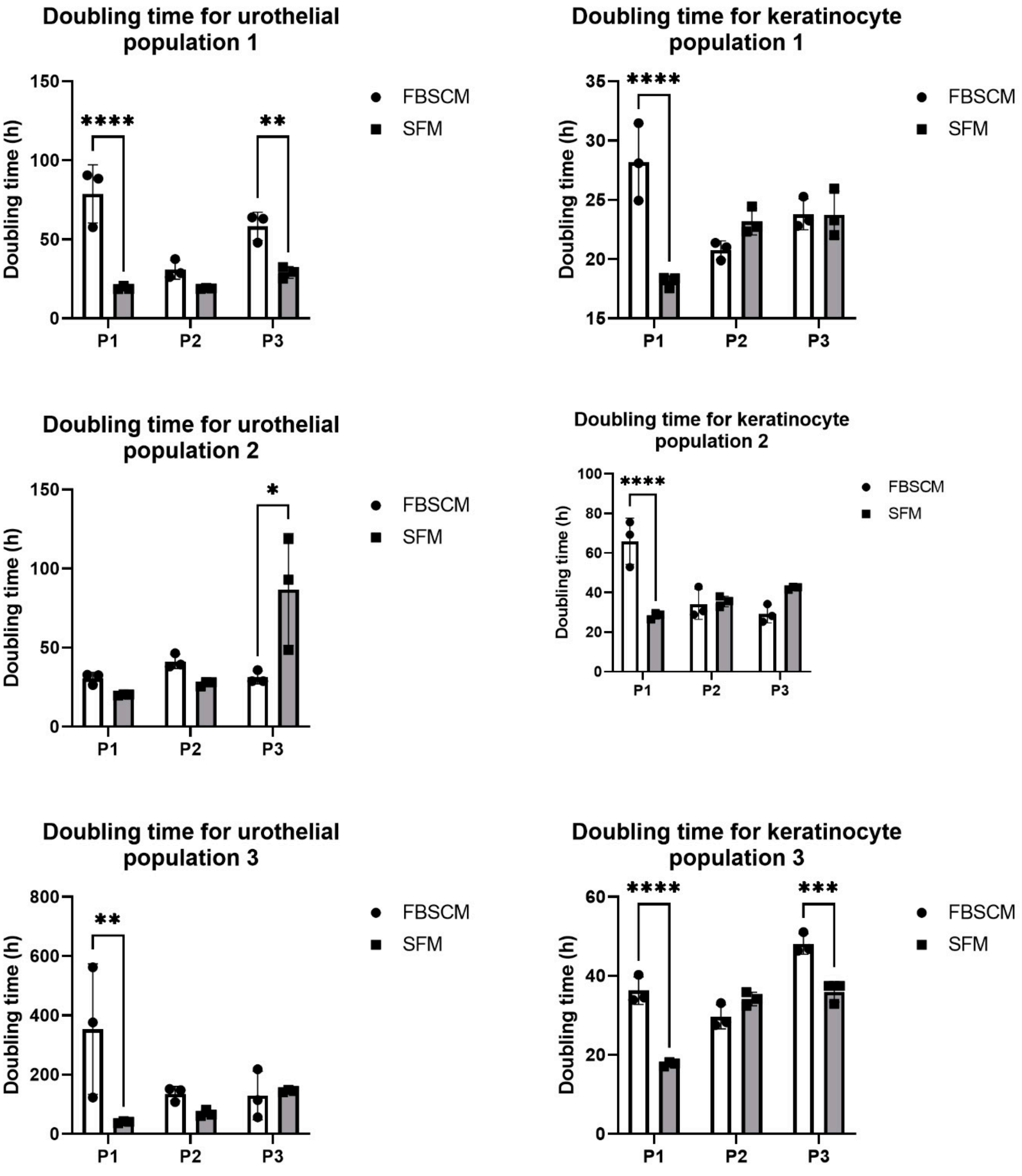

Figure S3B: Doubling time of fibroblast cells at passages 1, 2 and 3.

Doubling time of fibroblast cells over three passages. Ordinary two-way ANOVA has been used to interpret the data. Asterisks indicate significant differences: (*) for p-value<0.05, (**) for p-value<0.01. Each dot represents the mean of a cellular population realized in triplicate (n=3). Three points are presented as the experiment was done with three populations (N=3).

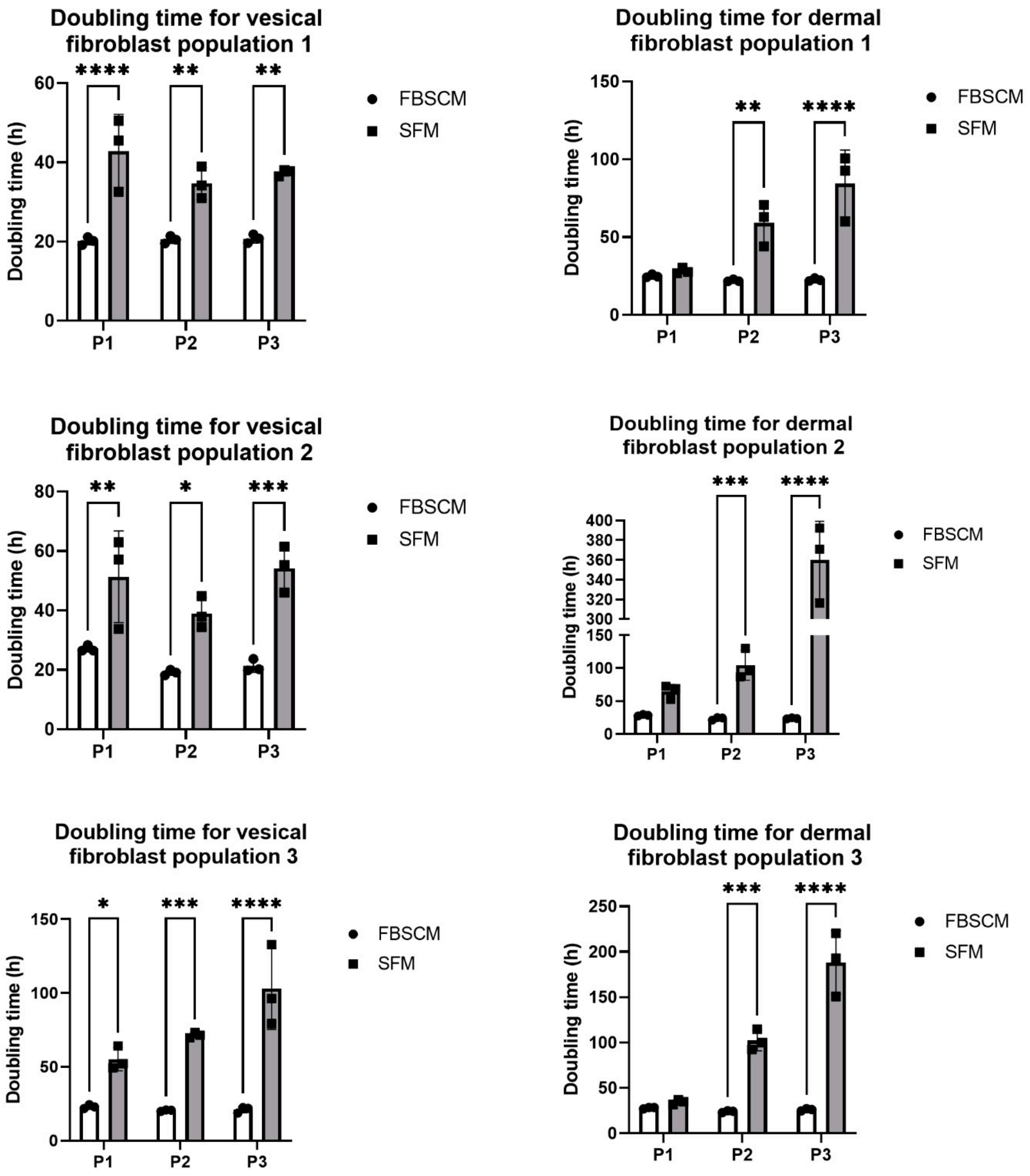
Doubling time for vesical fibroblast population 1
Doubling time (h)
60
40
20
0
P1
P2
P3
****
**
**
FBSCM
SFM
Doubling time for dermal fibroblast population 1
Doubling time (h)
150
100
50
0
P1
P2
P3
**
****
FBSCM
SFM
Doubling time for vesical fibroblast population 2
Doubling time (h)
80
60
40
20
0
P1
P2
P3
**
*
***
FBSCM
SFM
Doubling time for dermal fibroblast population 2
Doubling time (h)
400
380
360
340
320
300
150
100
50
0
P1
P2
P3
***
****
FBSCM
SFM
Doubling time for vesical fibroblast population 3
Doubling time (h)
150
100
50
0
P1
P2
P3
*
***
****
FBSCM
SFM
Doubling time for dermal fibroblast population 3
Doubling time (h)
250
200
150
100
50
0
P1
P2
P3
***
****
FBSCM
SFM

Figure S4: Macroscopical aspect of the flasks for evaluation of the CFUs.

Example of the macroscopic aspect of the same epithelial population in two flasks: (A) cell cultured in FBSCM and (B) cell culture in SFM.

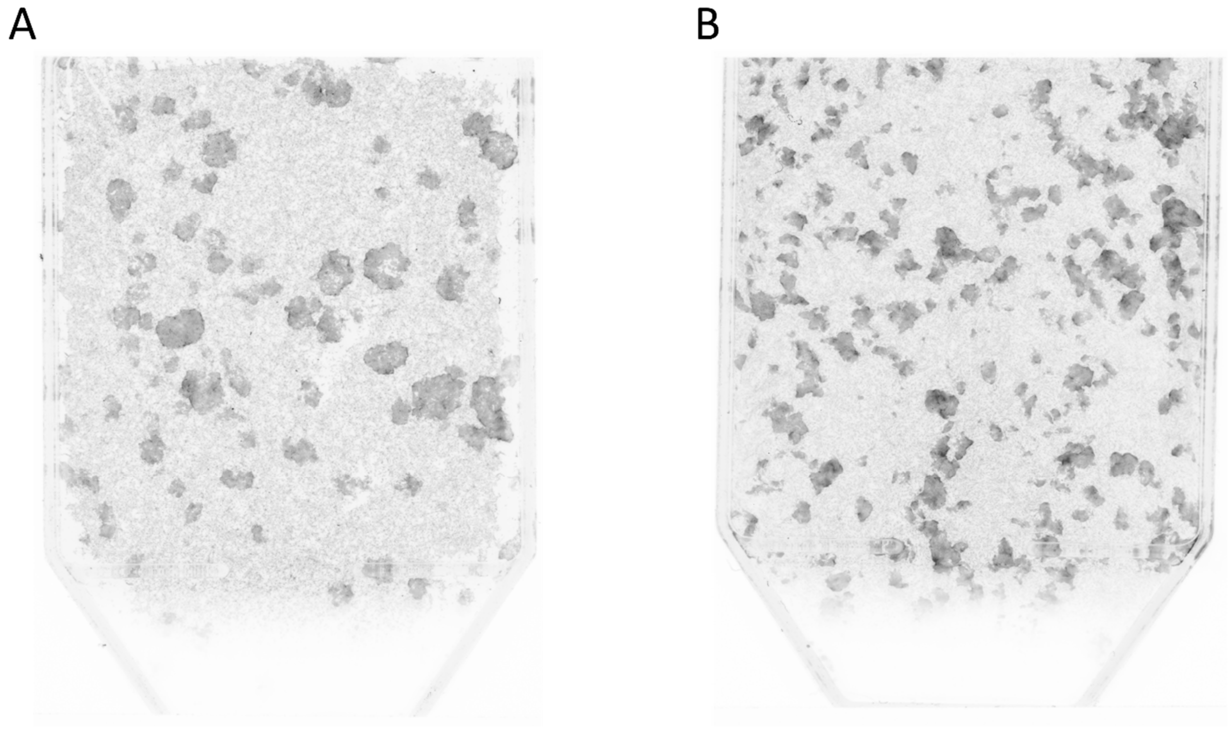

Figure S5A: Clonogenicity evaluation using the count technique.

Clonogenicity evaluation of keratinocytes (KC) and urothelial cells (UC) over three passages using the count technique. Ordinary two-way ANOVA has been used to interpret the data. Each dot represents the mean of a cellular population realized in triplicate (n=3). Three points are presented as the experiment was done with three populations (N=3).

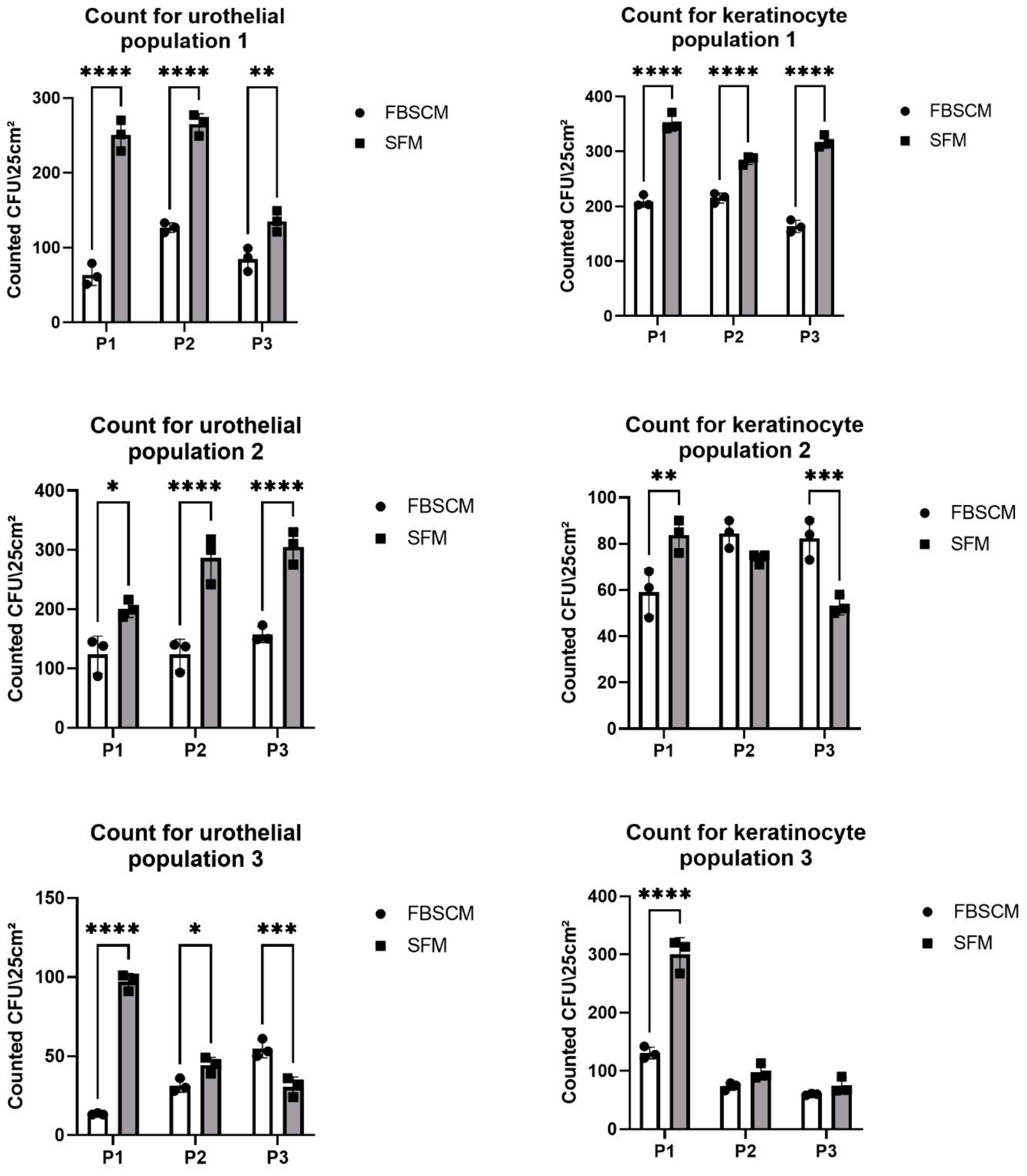

Figure S5B: Clonogenicity evaluation using the scanning technique.

Clonogenicity evaluation of keratinocytes (KC) and urothelial cells (UC) over three passages using the scanning technique. Ordinary two-way ANOVA has been used to interpret the data. Each dot represents the mean of a cellular population realized in

triplicate (n=3). Three points are presented as the experiment was done with three populations (N=3).

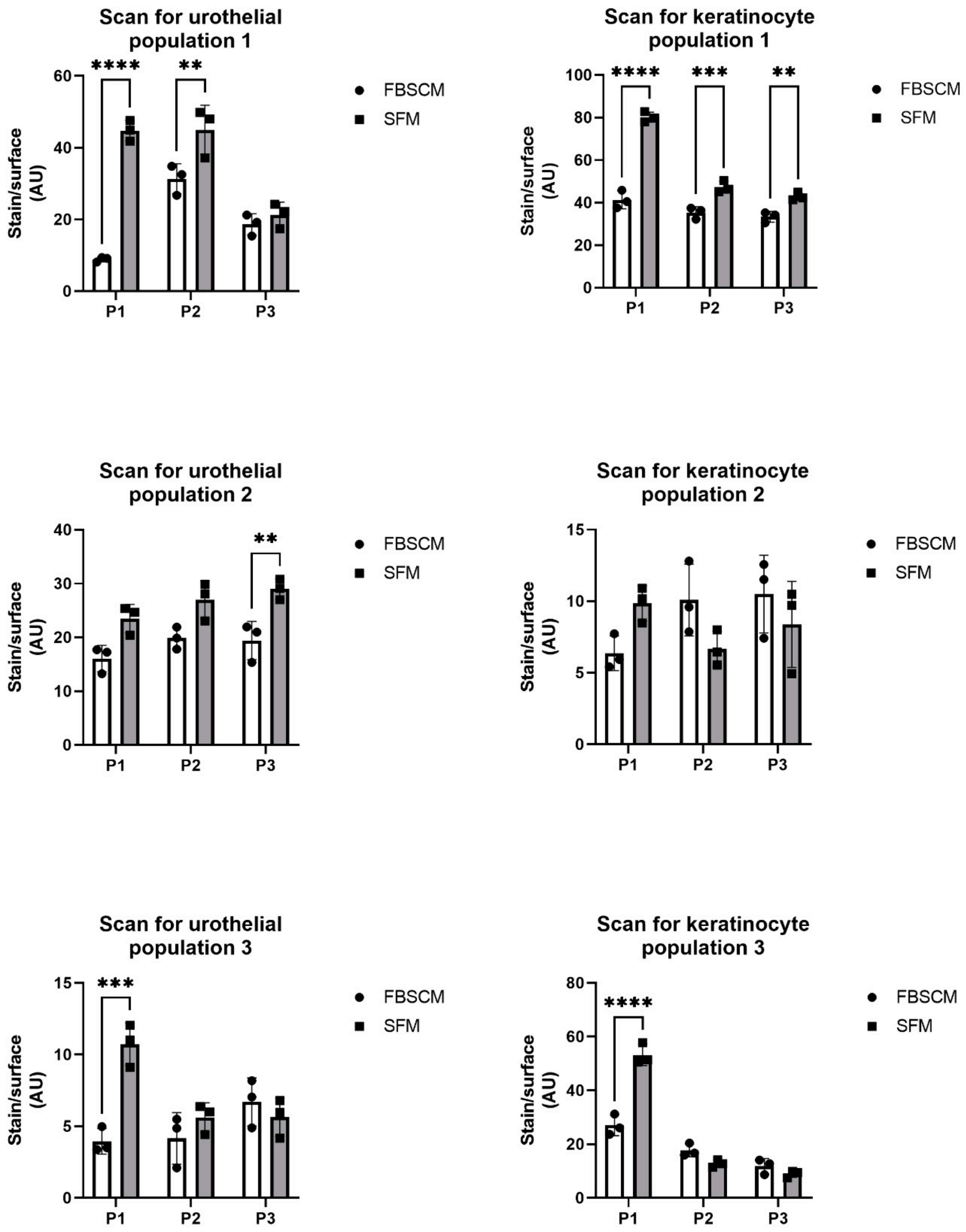

Figure S6: Extraction from biopsy and expansion to passage P0

Cell-count evaluation of keratinocytes (KC) after cell extraction and culture for six days, using the Coulter-Beckmann Z2 system. Four different experimenters have done the experimentation. Ordinary two-way ANOVA has been used to interpret the data. Each dot represents the mean of a cellular population realized in triplicate (n=3).

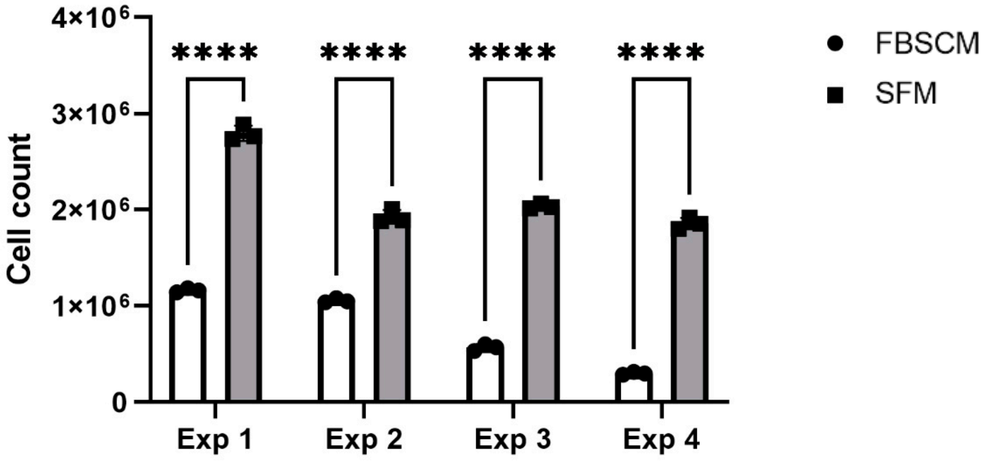

Supplement: Supplementary file 1 [file ijms-23-10035-s001.zip › ijms-1869814-supplementary.pdf]
